# Pasteur, Vaccines, and the Refusal to Become Fully Vaccinated in the Midst of the COVID-19 Pandemic

**DOI:** 10.3389/fpubh.2022.815816

**Published:** 2022-03-09

**Authors:** Charles S. Pavia

**Affiliations:** ^1^Department of Biomedical Sciences, NYIT College of Osteopathic Medicine, New York Institute of Technology, Old Westbury, NY, United States; ^2^Division of Infectious Diseases, New York Medical College, Valhalla, NY, United States

**Keywords:** COVID-19 vaccines, vaccine hesitancy, counter measures, public health, Pasteur

## Abstract

Vaccines are one of the most effective public health measures that are designed to prevent serious illness caused by a wide variety of infectious agents, which have become especially important in light of the coronavirus disease 2019 (COVID-19) pandemic. Despite the favorable outcomes associated with vaccine development and use, a new wave of hesitancy to get vaccinated has emerged that threatens the control or quick elimination of the highly contagious and life-threatening infection caused by SARS-CoV-2. At the forefront of the current anti-vaccine movement is the dissemination of false and misleading information. This essay explores the primary reasons, which also includes an historical connection, behind this anti-vaccine sentiment, and proposes several possible and realistic interventions that could be implemented to counter this notion and significantly improve vaccine acceptance, especially among young people.

## Introduction

The year 2022 marks the bicentennial of the birth of a man who probably changed the course of medical history and progress in the 19th century like no other person had done previously before the advent of modern medicine as we know it today. His name was Louis Pasteur—considered by many historians and scientists to be the founding father of modern microbiology and immunology. His discoveries laid the foundation for the many that followed that have led to improvements in hygiene, public health, and the prevention and the treatment of several diseases, primarily those having an infectious origin. In so doing, he paved the way for physicians to manage their patients better than they had done before due to the preventive measures that he developed (1). The many research and health care facilities that are scattered throughout the world bearing his name, are a testament to his justifiable fame in recognition of his ground-braking accomplishments.

Pasteur's life and scientific career, however, were not without their challenges, but his many successes, remarkable by today's standards, far outweigh whatever issues that he encountered. Pasteur was a chemist by training (as well as being a self-made and accomplished artist), and he established the use of the term “microbe,” based on his many discoveries and drawings/sketches of what he observed under the microscope. These findings often displeased some of his contemporaries, especially certain influential members of the medical establishment. Several of them were very reluctant initially to go along with some of his experimentally derived ideas and concepts, especially those pertaining to the use of vaccines as a preventive measure. This was based on them appearing to be quite radical at the time given the limitations of biomedical science and knowledge available during the mid-to-late 19th century. Despite possibly risking his career and reputation, Pasteur persevered working tirelessly to prove his doubters wrong who were either ignorant/less educated or prejudicial toward recognizing his ground-breaking discoveries. Indeed, he showed through careful experimentation that things not visible to the naked eye can cause disease, thereby establishing the germ theory of disease and paving the way for the future development of desirable vaccines. Eventually, many of his original detractors began to accept his revolutionary findings as being true.

## Discussion

With these historical accounts on Pasteur as the background, it is amazing and at times distressing to observe how certain aspects of this past scenario from another era are being re-visited today in the wake of the current COVID-19 pandemic. In this regard, many people, mostly outside the medical community, but a significant few within, have refused or remain hesitant to receive any of the available COVID-19 vaccines. Given the seriousness of COVID-19 with its high rate of mortality and the worldwide emergence of multiple highly contagious/virulent mutants, such as the Delta and Omicron variants, of the etiologic agent SARS-CoV-2, this reluctance to get vaccinated is almost unfathomable and has led to tragic results. In July 2021, as part of a nationally televised briefing (2), public health officials from the US Centers for Disease Control and Prevention (CDC) and the National Institutes of Health provided updates on a new surge nationwide in hospitalizations and deaths due to COVID-19, and have pointed out correctly that we were now entering into a “pandemic of the unvaccinated” which has subsequently continued into the months that followed. This comment by the director of the CDC is supported by data showing that in some locations >99% of the current wave of victims are among the unvaccinated.

Despite the high death toll due to COVID-19, why is there such hesitancy/refusal or impediments to getting vaccinated? The reasons are many and the major ones (summarized in Table 1) are as follows:

(a) People waiting to find out what will happen before deciding to take the vaccine. This is where vaccine recipients are observed for their response/reaction to the vaccines, in terms of making sure that the vaccines are safe and there will not be any negative outcomes; also, that the vaccines really work, or waiting for final approval for vaccine use from regulating agencies such as the US Food and Drug Administration (FDA) followed by further recommendations by the CDC;
(b) Fear of needles or being jabbed and having to endure a possibly painful injection. This is mostly a psychological or behavioral reaction that may have been triggered by prior unpleasant experiences when receiving injections for various other medications or from during a routine blood draw;
(c) Difficulty in gaining access to vaccine sites by the elderly, the severely disabled and impoverished people due to logistical complications;
(d) Exercise our constitutional rights. In the United States this claim is often cited by referring to the first amendment of the US Constitution. This view falls in line with not having to be compelled, similar to imposing a mandate, to take the vaccine as a requirement to participate in the activities associated with any organization, venue or event, such as a governmental agency, health-care facility, an academic institution, sport teams, transportation sites, houses of worship, indoor dining facilities, sponsors of entertainment or sporting events, or by an employer;
(e) Invoking an exemption based on “religious grounds” or spiritual upbringing;
(f) And perhaps the most flagrant of them all, believing or being swayed by the many forms of misinformation/disinformation or conspiracy theories. These include stating, too often in malicious tones, that the science behind the development of these vaccines and their success is fake, not to be trusted, vaccine recipients are being used as “guinea pigs,” or government recommendations are not to be trusted, These messages are being spread over certain Internet social media platforms and various radio and television cable news networks by mostly misguided or unscrupulous individuals, some of whom claim to have expertise on medical matters, but actually don't.

**Table 1:** Common reasons and their basis for COVID-19 vaccinees hesitancy/refusal.

**Reason**	**Basis or source of information**
Defer or postpone getting vaccinated	Vaccine needs to be fully approved by authorizing agencies such as the FDA and the CDC
Concern over serious reactions	Some serious adverse events have occurred in certain groups of vaccines
Anxiety/fear of being jabbed	Behavioral/psychological response based on prior unpleasant experiences
Unable to reach vaccine sites	Transportation issues or infrastructure problems
Personal freedom to choose or decide	Political convictions/stance protected by the US Constitution
Moral or religious beliefs	Influence of certain religious teachings or religious leaders (clergy) based on a skewed interpretation of “God-given” rights or principles
Vaccines are not safe or effective and distrust of government recommendations	Misinformation/conspiracy theories spread through various Internet social media sites and broadcast outlets by unqualified, non-scientific/non-medical commentators

It is important to realize that the anti-vaccine sentiment is not limited to the US, but it is a global problem (3) and had been well-established prior to the onset of the current pandemic. Evidence in support of the pervasiveness and the immediate effects of these untenable reasons against vaccines comes from some recently published statistics put forth by the Kaiser Family Foundation (4), and these are as follows.

In terms of demographics, 27% of the people in the United States remains vaccine-resistant, saying they probably or definitely would not get a COVID-19 vaccine even if it were available for free and deemed safe by scientists and public health officials. Vaccine hesitancy is highest among those who identify as belonging to a certain political party (42%), those in the 30–49 age group (36%), and rural residents (35%), especially those living in certain parts of the Midwest and Southeast where <50% of the population has gotten vaccinated, and where, concurrently, there has been a new surge (as of July/August 2021) in COVID-19 cases requiring hospitalization. In addition, 35% of African American adults (a group that has had to bear a disproportionate burden of the effects of the pandemic) say they definitely or probably would not get vaccinated, citing as major reasons that they don't trust vaccines in general (47%) or that they are worried they may get sick with COVID-19 from just the vaccine alone (50%). This situation suggests that messages combatting particular types of misinformation may be especially important for increasing vaccine confidence among this group. Perhaps most astonishingly, are the data showing that as much as one third of those who say they are considered to be essential workers and 29% of those who perform services in a health care delivery setting will not take the vaccine (4). Such hesitancy in this latter group has been re-affirmed and detailed by another group of investigators in a recently published review article (5). These authors reported that the prevalence of COVID-19 vaccination hesitancy worldwide in healthcare workers ranged from 4.3 to 72% (average = 22.51% across all studies with 76,471 participants). In the majority of cases there were concerns about vaccine safety, efficacy, and potential side effects as top reasons before agreeing to get vaccinated against COVID-19, mostly because the effort to produce the vaccines was seen as being too hasty or too much of a speedy and less rigorous process, and having also political overtones. Some workers also felt that, as part of their work environment in caring for patients, they probably had already been well-exposed to the virus and had developed protective antibodies as a result of some form of “natural immunity.” Accordingly, any subsequent anti-COVID vaccine would be deemed as being unnecessary and/or could lead to yet-to-be-determined adverse reactions. Interestingly, on the other hand, it was found that the majority of those who were males, of older age, and clinicians were more likely to accept COVID-19 vaccines. As a result of such hesitancy, it is noteworthy that, beginning with New York State in September 2021, and then followed by other jurisdictions, including agencies within the United States federal government, COVID-19 vaccine mandates were enacted for medical workers and many other work environments. However, these have already met legal/judicial challenges for their implementation and may cause a workforce crisis at some hospitals and nursing homes, and various other facilities. In marked contrast and perhaps somewhat unexpectedly, it has been shown (6) that, in some locations, COVID-19 has increased acceptance of influenza vaccination in previously eligible but unvaccinated people and has motivated substantial uptake in newly eligible people.

Pasteur's anniversary offers a time to reflect on what he might advocate to encourage greater vaccine acceptance short of issuing mandates. In this regard, the CDC has made numerous suggestions (7), similar and compatible to what this author also now proposes and which is in keeping with suggestions made by an expert panel of biomedical investigators (8) that “we bring forward a novel solution and change the status quo.” Accordingly, such reluctance may require that more intense, frequent and innovative approaches need to be taken. They include several measures aimed primarily at educating young people, especially students, and their families in the right direction. Initially, along these lines, a large number of healthcare professionals, especially infectious disease specialists, and qualified scientists (primarily microbiologists and immunologists) are needed to support COVID-19 vaccination efforts and strategies nationwide and beyond. It is important that they are adequately trained to effectively meet the demands of their roles. Training must be continuous and updated as new or modified COVID-19 vaccines are developed, as vaccine policies evolve, such as whether additional booster shots are needed, and more is learned about the vaccines, and how to improve and maintain the vaccination process. In terms of educating the general public, these qualified personnel are essential to ensuring that people get vaccinated safely as soon as possible, based on a true understanding on why this is an important undertaking. Furthermore, as a trusted source of information for parents, pediatric and adolescent healthcare professionals play a critical role in helping parents and guardians realize the importance of receiving a vaccine and assuring them that COVID-19 vaccines are safe and effective, and that they are important steps in protecting their eligible children's health (both physical and mental) as well as other family members. Parents also need to understand that fully vaccinated people are less likely to spread the virus that causes COVID-19. Getting all family members 5 years and older fully vaccinated can protect other family members especially the elderly or others with pre-existing health conditions who are part of a group at increased risk for developing severe illness from COVID-19 (9). Pre-college students also need to realize that, after they are fully vaccinated, they will be able to resume many activities that they have missed due to prior restrictions that were imposed at the height of the pandemic.

As an additional approach, schools, at both the elementary and high school level, can also take the initiative by recruiting the suitably trained medical professionals or scientists to come to their classrooms and supplement the curriculum by providing a better understanding of the vaccine process. In order to accomplish this task more effectively, local health departments, especially those within the jurisdiction of the schools, should provide the schools with a registry of trained personnel who would be willing to come to the schools and share their knowledge and expertise pertaining to the COVID-19 vaccines, as well as answering any questions or concerns that students may have on this topic, preferably at no additional cost to the school district.

As part of the education process, other related steps can be taken including periodically contacting students and encouraging them to learn more from reputable sources derived from articles (especially those that are peer-reviewed), social media and blog sites, properly mentored student-driven publications and affiliated groups. Beyond the classroom setting when it resumes fully, a feedback mechanism should be established in order for students to ask questions to, and get a reliable response quickly from, the qualified members of the health care and scientific community about COVID-19 vaccination, using either e-mail, online video conferences, such as via Zoom or Facetime, or by direct phone calls/text messages. Any student concerns or questions should be proactively addressed and damage caused by the spread and harm of misinformation and, in some cases, outright deception should be countered by sharing credible and accurate information. Students should be made aware about misinformation and disinformation, and health literacy should be stressed as a way to be fully informed in understanding the benefits of being vaccinated as well as the negative outcomes that could happen by not getting the vaccine. Although much of the preceding suggestions pertain mostly to the adolescent age group, similar interventions should also be considered for those attending colleges and universities after completing high school, to reinforce and update what students had learned previously about the vaccine, or for those college students who did not have the same type of initial learning experience. Hopefully, as this younger and now well-informed generation matures into adulthood, they will be representative of a population having much greater acceptance and less resistance to getting vaccinated when deemed necessary by experts in the health care community for both now and, just as important, later on, when other future serious outbreaks may arise.

## Conclusion

To promote much wider and long-term acceptance of COVID vaccines and other life-saving vaccines, major changes need to be considered. Many of the proposals outlined in this perspective have focused on improving the way that the scientific and biomedical community can better educate the general population on vaccine development and the advantages of getting vaccinated in the face of vaccine hesitancy/refusal. The emphasis is placed on making this a more intense and focused educational approach that would be a coordinated effort involving regional health agencies, school districts, and students and their family members. It is important that this process be tested and periodically evaluated to ensure its success. Consistent with these proposed interventions, Pasteur often hinted at possibilities of future discoveries, which later became accomplished facts (1). Perhaps we can learn again from such lessons from the past where there is a re-awakening of the public interest in preventive medicine where it can be shown that, in dire circumstances, life-saving vaccinations are not something to be feared but should be embraced as absolute necessities.

## Data Availability Statement

The raw data supporting the conclusions of this article will be made available by the authors, without undue reservation.

## Author Contributions

The author confirms being the sole contributor of this work and has approved it for publication.

## Conflict of Interest

The author declares that the research was conducted in the absence of any commercial or financial relationships that could be construed as a potential conflict of interest.

## Publisher's Note

All claims expressed in this article are solely those of the authors and do not necessarily represent those of their affiliated organizations, or those of the publisher, the editors and the reviewers. Any product that may be evaluated in this article, or claim that may be made by its manufacturer, is not guaranteed or endorsed by the publisher.
